# The Role of Stable Anatomical Landmarks in Automated 3D Model Superimposition: A Closer Look

**DOI:** 10.3390/bioengineering12080839

**Published:** 2025-08-03

**Authors:** Tommaso Castroflorio, Samuele Avolese, Fabrizio Sanna, Simone Parrini

**Affiliations:** 1 Clear Aligner Academy, 10100 Torino, Italy; 2 Department of Mechanical and Aerospace Engineering, Polytechnic University of Turin, 10100 Torino, Italy; 3 Department of Surgical Sciences, Dental School of the University of Torino, 10100 Torino, Italy

**Keywords:** dental cast superimposition, digital models, palatal rugae, orthodontic tooth movement accuracy

## Abstract

Objective: To evaluate the concordance of automated 3D superimposition methods applied to digital models, with a focus on methods that consider stable palatal regions as geometric reference landmarks versus those that do not. Design and setting: This was a prospective, cross-sectional study using digital model files of patients undergoing orthodontic treatment in a university clinical setting. Participants: Sixty-one patients were prospectively enrolled and divided into three groups based on the type of orthodontic treatment they received: (20) non-extractive orthodontic treatment without intermaxillary elastics, (21) intermaxillary elastics, and (20) control subjects with no orthodontic movement. The inclusion criteria included the availability of complete pre- and post-treatment digital casts and the absence of significant craniofacial anomalies. Methods: Three superimposition methods were tested: (1) superimposition according to palate and palatal ridges, (2) best-fit superimposition of arches in occlusion, and (3) best-fit superimposition of individual arches. Discrepancies were identified by comparing the spatial positions derived from each method. Within three spatial axes, deviations of ±0.5 mm and ±1.15° were not considered significant. Bland–Altman plots were used to quantify palatal rugae based and non-based spatial differences between methods. Differences in the superimposition results between the three patient groups were evaluated using ANOVA tests. Results: Differences in spatial position between the superimposition methods often exceeded the acceptable range. The results were compared between the three patient groups with a statistical significance of α = 0.05. In the present study, the high reliability of the superimposition method based on the palate and palatal ridges was observed. Conclusion: Superimposition methods based on the palate and palatal rugae provide superior accuracy in determining treatment-related changes in upper arch digital models. These findings illustrate the need for appropriate selection of superimposition techniques based on the study objective of using clinically relevant techniques.

## 1. Introduction

Orthodontists have traditionally relied on 2D lateral cephalometric radiographs for both treatment outcome evaluation and growth assessment [1,2,3]. Superimposing pre- and post-treatment radiographs to assess changes in dental and skeletal structures is a common modality used in this method. However, lateral cephalometry has inherent drawbacks, including the potential for bias associated with head positioning and distortion of 3D structures represented in 2D [4,5].

In recent years, the use of cone-beam computed tomography (CBCT) superimposition has been proposed as an alternative [6,7,8]. CBCT provides an accurate assessment of maxillary changes through the identification of craniofacial landmarks and facilitates the superimposition of dental structures before and after treatment, thus allowing the assessment of orthodontic predictability [9]. Although CBCT has great potential, it is not a standard diagnostic tool and must be used according to guidelines and regulations [10].

Superimposing three-dimensional (3D) digital models has clear advantages, including no radiation exposure, high accuracy, and elimination of distortions introduced by the radiographic process. However, although cuspal position can be superimposed, stable anatomical landmarks are challenged by changes in growth or extraction space closure during treatment [11].

The palate is considered a consistent reference structure to obtain accurate superimposition of 3D models of the maxillary arch [6]. The stability of palatal landmarks during orthodontic treatment with or without miniscrew reference points was compared with classic lateral radiographs [12,13,14,15,16,17]. The aforementioned landmarks, including the medial half of the third palatal rugae and the mid-palatal raphe, have been established in five-year longitudinal studies and can, therefore, serve as stable landmarks [18,19,20,21,22].

In contrast, repetitive static mandibular landmarks are not well characterized, except for the mandibular torus, which is missing in some patients [23]. To solve this problem, a relative position of the mandible can be obtained by superimposing the maxillary arch, activating an indirect relationship of the mandibular arch with the palate [17].

As a result, numerous software applications have been developed to facilitate the automated superimposition of digital models, taking advantage of the AI algorithms used to increase the accuracy of registration and measurements. The accuracy, cost, and ease of use of these devices vary [24,25]. Technical variables also affect the accuracy of digital model superimposition. Automated superimposition algorithms, including iterative closest point (ICP) or surface-based registration methods, can be easily compromised by noisy data, low-resolution models (below 270 microns), or matching of inappropriate regions [26,27,28,29,30]. Superimposition-based methods often exclude soft tissue, as this can further bias the results [31], especially during tooth extraction, as mandibular space closure has been shown to alter the morphology and consequently the positions of landmarks [11]. Manual landmark identification also introduces inter- and intra-observer variability, compromising reproducibility [9,13]. These findings suggest that further research is needed to optimize superimposition methods, validate computerized systems, and establish standards for clinical application.

To date, no prospective clinical study has compared different 3D superimposition methods for both maxillary and mandibular arches in growing and non-growing patients using digital model analysis.

Therefore, the aim of this study was to compare the reproducibility of automated digital 3D superimposition approaches that use anatomically stable palatal landmarks with those that do not.

The primary null hypothesis was that there would be no significant difference in the accuracy of model superimposition among the three techniques tested: superimposition on palatal ridges, best-fit superimposition of arches in occlusion, and best-fit superimposition of separate arches.

The secondary null hypothesis was that no significant differences would be observed between the three study groups:

(1) Individuals treated with aligners without extraction, intermaxillary elastics, or skeletal anchorage;
(2) Patients treated with intermaxillary elastics in conjunction with clear aligners for non-extraction Class II correction without skeletal anchorage; and
(3) Untreated controls.

## 2. Materials and Methods

### 2.1. Subjects

This prospective study analyzed digital models of 61 patients consecutively recruited at the Department of Orthodontics of the University of Torino, between June 2018 and July 2022. Participants were selected based on predefined inclusion and exclusion criteria, using an alphabetical list of eligible individuals. To ensure unbiased allocation, a blinded investigator randomly determined the selection interval. The study was conducted according to the Guidelines for Reporting Reliability and Agreement Studies (GRRAS) [32] and received ethical approval from the Ethics Committee of Città della Salute e della Scienza di Torino (no. 157/2020). Written informed consent was obtained from all the participants before enrollment, and all the procedures adhered to the ethical principles of the Declaration of Helsinki of 1964 and its subsequent amendments.

The participants were categorized into three groups:

1. Group 1 (G1): A total of 20 patients (6 males and 14 females; mean age 25.6 years; mean treatment duration 544 days) treated with clear aligners, without intermaxillary elastics, skeletal anchorage, or extractions.
2. Group 2 (G2): In total, 21 patients (4 males and 16 females; mean age 16.38 years; mean treatment duration 615 days) treated with clear aligners, involving class correction with intermaxillary elastics but no extractions or skeletal anchorage.
3. Group 3 (G3, Control): A total of 20 untreated individuals (9 males and 11 females; mean age 25.48 years; mean interval 576 days).

All the subjects included in the study presented with either Class I or end-to-end Class II Division 1 malocclusion.

The inclusion criteria for the study required the participants to have all permanent teeth present (except third molars), fully erupted second molars, and an intact palatal vault on both the initial (T0) and final (T1) intraoral scans. The exclusion criteria excluded individuals with a history of temporomandibular disorders or craniofacial trauma, syndromic conditions, scans showing hard or soft tissue abnormalities, the presence of prosthetic restorations, or a history of periodontal disease, and those with prior orthodontic or orthopedic treatment.

### 2.2. Records

Intraoral scans were obtained with the iTero Element 2 scanner (Align Technology, Inc., San Jose, CA, USA). The scans were performed by orthodontic residents who supervised patient care and were blinded to the study to minimize bias. The scanns has been used to avoid the errors that can be incurred by scanning with a laboratory scanner an analogic model previously cast in plaster [33].

All the scans were securely stored on a terminal computer connected to a cryptographically secured server with access restricted to the principal investigator. The scans were acquired at two time points: treatment initiation (T0) and treatment completion (T1). The scans were then de-identified and exported as .stl files. These files were analyzed using specialized software (3D Slicer (CMF 5.0, www.slicer.org (accessed on 7 July 2025)), Geomagic Control X (version 2020.1.1, 3D Systems, © Inc., Rock Hill, SC, USA) and Viewbox 4 (version 4.1.0.1 BETA, dHAL Software, Kifissia, Greece), by an experienced operator at the Department of Orthodontics, University of Torino.

### 2.3. Superimposition Techniques

The T0 and T1 .stl files were superimposed using three different methods using two specialized software programs. Method 1 used 3D Slicer (CMF 5.0, www.slicer.org (accessed on 7 July 2025)) to identify palatal ridges as stable landmarks. Methods 2 and 3 used the best-fit algorithm in Geomagic Control X (version 2020.1.1, 3D Systems, © Inc., Rock Hill, SC, USA), which aligns 3D models by minimizing surface distances without requiring an initial global alignment. The reliability of this approach has been validated by Stucki and Gkantidis [19]. Of note, all the methods were consistently applied by an experienced operator using the same digital models.

The origin of the coordinate system varied between the methods: method 1 used the medial palatal raphe as the central reference defined by fiducial points, whereas methods 2 and 3 used the geometric centroid of the dental arches as the spatial origin. These origins were maintained within each method to ensure consistent analysis.

**Figure 1:**
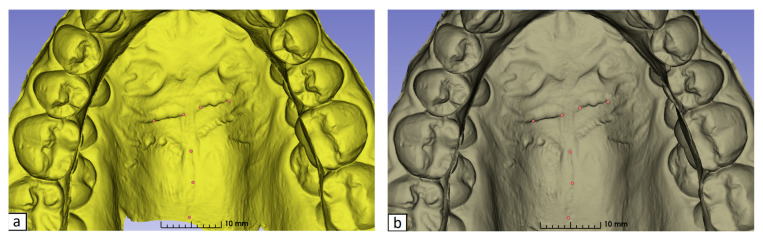
Identification of fiducial points on T0 (**a**) and T1 (**b**) scans.

The identification of the palatine rugae (method 1) was performed using 3D Slicer, as previously described by Anacleto et al. [34]. Seven fiducial points, as reported by Vasilakos et al. [18], were marked: the medial ends and midpoints of the third palatal ridges bilaterally, and three points along the midpalatal raphe (the midpoint of the fourth palatal ridge, a point 5 mm posterior, and another 10 mm posterior) (Figure 1).

**Figure 2:**
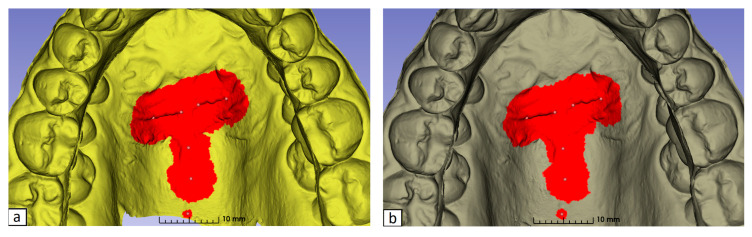
ROI selection on T0 (**a**) and T1 (**b**) scans.

The “Surface Registration/ROI Registration/Add and Move Landmarks” function in 3D Slicer was utilized to facilitate this process. The region of interest (ROI) sizes were set at 30 mm^2^ for the third palatal ridges, 20 mm^2^ for the first two midpalatal raphe points, and 5 mm^2^ for the third point, based on studies highlighting these regions’ stability and precision [18] (Figure 2).

**Figure 3:**
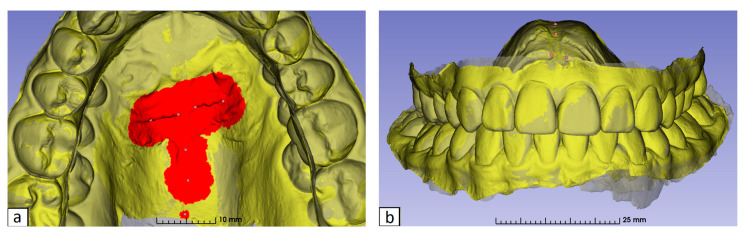
Superimposition on the identified ROI from the occlusal (**a**) and frontal (**b**) view.

**Figure 4:**
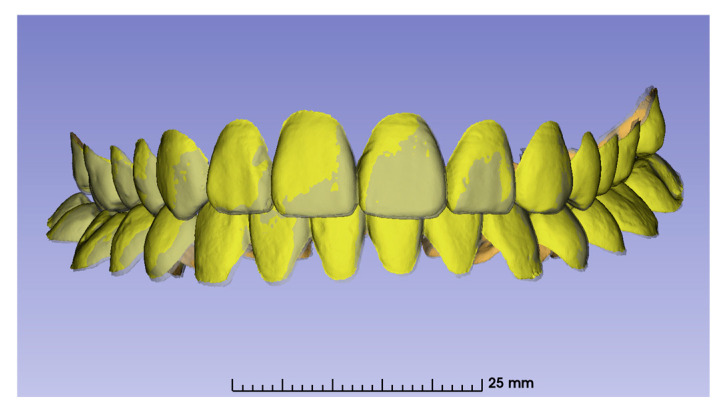
Virtual soft tissue removal after ROI overlay.

**Figure 5:**
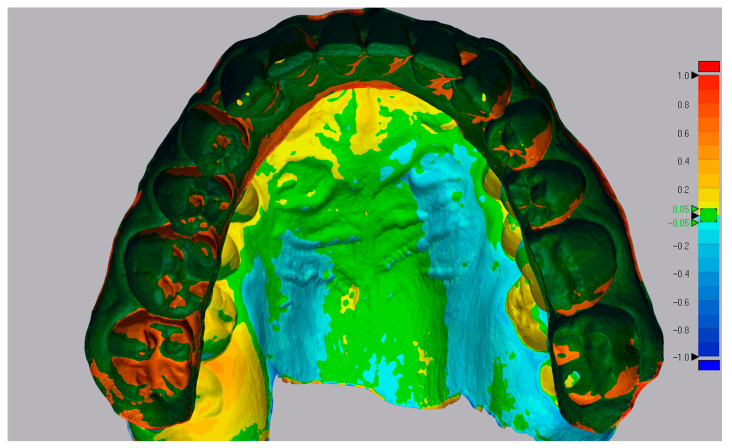
Color map showing how well palatal rugae region overlaps.

The T0 model was designated as the fixed reference, and the T1 model was aligned to it using a best-fit algorithm applied to the selected ROI (Figure 3). The removal of soft tissue (Figure 4) and the overlay comparison (Figure 5) were performed using the Geomagic Control X software (version 2020.1.1, 3D Systems, © Inc., Rock Hill, SC, USA).

**Figure 6:**
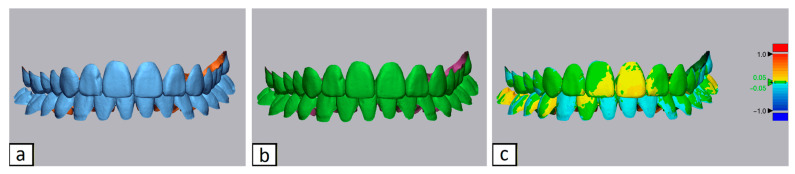
Superimposition using method 2. Control group patient. From left to right: scan at T0 (**a**), scan at T1 (**b**), and color map of the best-fit superimposition of the arches in occlusion (**c**).

The best-fit superimposition of arches locked in occlusion (method 2) was performed by importing the .stl files of both arches in occlusion into Geomagic Control X. After soft tissue removal, the “Best-Fit Alignment” function automatically superimposed T1 scans onto T0 scans for comparison (Figure 6).

**Figure 7:**
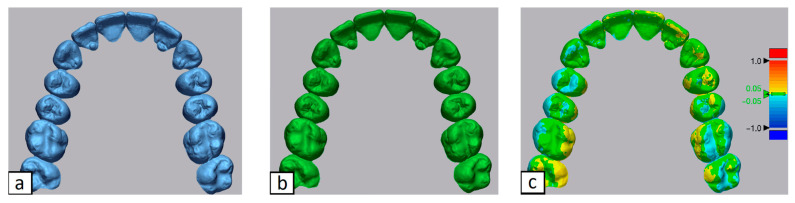
Overlay using method 3, upper arch. Control group patient. From left to right: scan at T0 (**a**), scan at T1 (**b**), and color map of the best-fit superimposition of the single upper arch (**c**).

**Figure 8:**
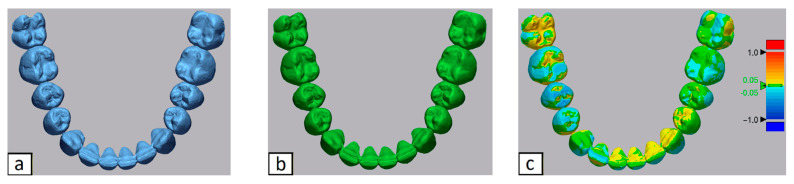
Overlay using method 3, lower arch. Control group patient. From left to right: Scan at T0 (**a**), scan at T1 (**b**), and color map of the best-fit overlay of the single lower arch (**c**).

The best-fit superimposition of separate arches (method 3) was obtained by uploading the T0 and T1 .stl files for each separate arch into Geomagic Control X. Following the removal of soft tissue, the upper arches at T0 were aligned with corresponding upper arches at T1 using the “Best-Fit Alignment” function and similarly, the lower arches at T0 were aligned with the corresponding lower arches at T1 (Figure 7 and Figure 8). 

In methods 2 and 3, we used the “selected data” sub-option in the best-fit superimposition to focus the alignment process on selected data regions. In this way, irrelevant areas such as variability-prone regions (i.e., teeth without full scans) were ignored during alignment, which in turn could lead to more accurate results. For the best-fit superimposition, a maximum number of 30 interactions [28], a sampling ratio of 25% and no movement constraint in the three coordinates were used for the settings; no manual adjustment was made afterwards, and no reference structure or symmetry assumption was employed.

### 2.4. Measuring Differences in Superimposition Methods

After completing all the superimpositions, measurements were collected, focusing solely on the fit of the dental arches without including soft tissues. Differences between the superimpositions of methods 2 and 3 were then calculated in comparison to method 1. The superimposed .stl files from Geomagic Control X were exported as single files for each superimposition, with updated spatial coordinates for the T1 model relative to the T0 reference. These single .stl files were then imported into Viewbox 4 (version 4.1.0.1 BETA, dHAL Software, Kifissia, Greece) for further analysis of spatial displacements and rotational differences. The three-dimensional displacement of the models from methods 2 and 3 relative to the model from method 1 was analyzed using the Viewbox 4 software. Data were expressed in millimeters for translations along the three spatial axes and in degrees for rotations around these axes. For consistency, the x-axis represents the latero-lateral (right–left) axis, the y-axis represents the sagittal (anterior–posterior) axis, and the z-axis represents the vertical axis. Linear measurements along the x, y, and z axes indicate the accuracy of methods 2 and 3 in identifying the correct right–left, anterior–posterior, and vertical positions of the T1 model, respectively, when compared to method 1. Similarly, angular measurements along the x, y, and z axes reflect the accuracy of methods 2 and 3 in identifying the roll, pitch, and yaw (rotation) of the T1 model, respectively, compared to method 1.

### 2.5. Statistical Analysis

The required sample size was based on previous research investigating the accuracy of software for estimating 3D orthodontic tooth movement [24,35]. Based on these findings, 20 scans were considered sufficient for this agreement study, with a minimum acceptable reliability (*ρ*_0_) of 0.60, a maximum expected reliability (*ρ*_1_) of 0.90, and *κ* = 3 (representing the number of superimposition methods). The statistical significance level (*α*) was set at 0.01, and the power (1 − *β*) at 0.9. Although the initial calculation indicated a minimum sample size of 18, this was increased to 20 to account for any scans of insufficient quality.

All the measurements were recorded in a Microsoft Excel^®^ spreadsheet (Microsoft, Redmond, WA, USA) and analyzed using the SPSS^®^ statistical software, version 24 (IBM Corporation, Armonk, NY, USA). Variations greater than ±0.5 mm were considered significant when comparing spatial displacements between methods 2 and 3 relative to method 1 across the three spatial axes [26,36]. For rotational differences, variations exceeding ±1.15° were considered significant. The threshold of ±1.15° was established based on the calculation that a rotation of this magnitude for the entire arch would result in a spatial displacement of 0.5 mm at the molar level, given an average intermolar distance of 50 mm (radius 25 mm) [37], calculated as follows:

Arc Length = Radius × Angle (in radians)  ⟹  25  mm × 1.15° × (π/180) ≈ 0.5 mm

To evaluate the discrepancies in spatial position between methods 2 and 3 compared to method 1, Bland–Altman plots were used, with a 95% confidence interval for the mean differences. The null hypothesis assumed that the confidence interval limits would fall within ±0.5 mm for positional differences and ±1.15° for rotational differences. Spatial position values outside these limits were considered significantly different. For method 3, positional differences were calculated and analyzed separately for the upper and lower arches.

ANOVA tests with nonparametric Friedman’s repeated measures were conducted to assess the presence of significant differences between the medians of spatial positions detected by the different superimposition methods. These tests compared changes in the same spatial position variable across methods 2 and 3. If significant differences were found, post hoc tests were applied to identify the source of the differences, with this procedure repeated for each group.

Since the range of differences between superimposition methods relative to the standard can include both positive and negative values, the absolute values of these differences were used in the analysis. This was performed to avoid a median close to zero, which could obscure statistically significant differences [26].

A linear regression analysis was then performed on the control group data to test whether variables such as sex, age at T0, and the time elapsed between T0 and T1 could influence the superimposition results. Additionally, a linear regression was conducted on the absolute values of the positional differences between method 1 and methods 2 and 3 to determine the absolute error between the different superimposition methods. The regression analysis was limited to the control group, as this group was the only one without orthodontic treatment, thus providing a homogeneous dataset. For all the tests, statistical significance was set at *p* < 0.05.

## 3. Results

The age distribution within each group was as follows: Group 1 (mean ± SD: 25.6 ± 3.8 years, range: 20–32), Group 2 (16.38 ± 2.1 years, range: 14–19), and Group 3 (25.48 ± 4.2 years, range: 21–30).

All the superimpositions and measurements across the three software platforms were conducted by a single experienced operator. Intra-rater variability was assessed by repeating measurements on a subset of 10 randomly selected models, with consistency evaluated using the intraclass correlation coefficient (ICC). The ICC values exceeded 0.95 for all measurements, indicating excellent consistency.

**Scheme 1:**
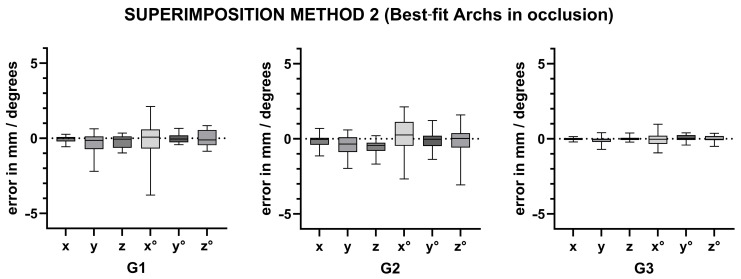
Visual assessment of the discrepancy between the superimposition methods 2 and 1.

**Scheme 2:**
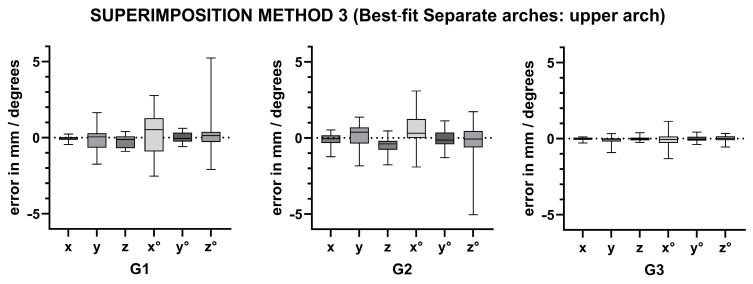
Visual assessment of the discrepancy between the superimposition methods 3 (upper arch) and 1.

**Scheme 3:**
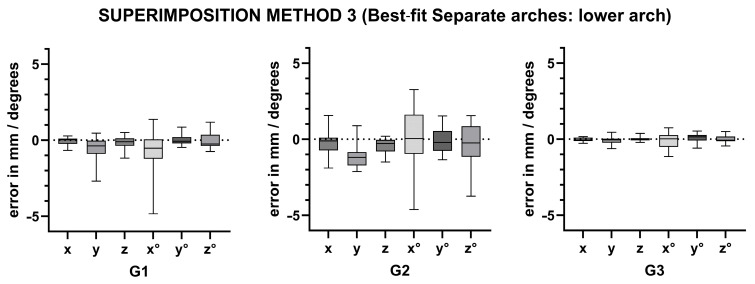
Visual assessment of the discrepancy between the superimposition methods 3 (lower arch) and 1.

The Bland–Altman analysis revealed that the null hypothesis was supported for some of the positional and rotational differences across the three spatial axes among the different superimposition methods. However, in other cases, the differences exceeded the predefined threshold values (±0.5 mm for positional differences and ±1.15° for rotational differences). Thus, while the three superimposition methods demonstrated equal accuracy in positioning the T1 models in space for certain positions and rotations, they showed significant discrepancies for others (Table 1, Table 2 and Table 3 and Scheme 1, Scheme 2 and Scheme 3).

In Table 1 the values in bold emphasize the importance of selecting appropriate superimposition methods depending on the clinical context. While method 2 (Best-Fit Alignment) may work for broader comparisons, it appears less reliable for precise measurements in cases where rotational or translational accuracy is critical.

In Table 2 the results in bold suggest that method 3 (Best-Fit Separate Arches: Upper Arch) introduces greater variability compared to method 1 due to the lack of reliance on stable anatomical landmarks. While method 3 may suffice for broader assessments, it is less suitable for applications requiring high precision, such as evaluating subtle tooth or skeletal changes.

The bold values reported in Table 3 indicate that method 3 (Best-Fit Separate Arches: Lower Arch) introduces greater variability compared to method 1, particularly for precise measurements of translational and rotational changes. These discrepancies suggest that method 3 is less reliable for detailed assessments of mandibular movements or rotations.

The ANOVA test comparing superimposition methods 2, 3 (upper jaw), and 3 (lower jaw) revealed statistically significant differences in a few cases. Specifically, the following results were revealed:

- In Group 1, a significant difference was found in translation along the x-axis (*p* = 0.022).
- In Group 2, significant differences were observed in translation along the x-axis (*p* = 0.01) and y-axis (*p* < 0.001), as well as in rotation around the x-axis (*p* = 0.041) and y-axis (*p* = 0.013).
- In Group 3, a significant difference was found in translation along the x-axis (*p* = 0.01).

Pairwise comparisons were performed to identify which methods caused these differences. The key findings were as follows:

- Group 1: A significant difference in translation along the x-axis was found between method 2 and lower jaw method 3 (*p* = 0.034).
- Group 2: Significant differences were found in the following:
- Translation along the x-axis between method 2 and lower jaw method 3 (*p* = 0.01).
- Translation along the y-axis between method 2 and lower jaw method 3 (*p* = 0.001), and between upper jaw method 3 and lower jaw method 3 (*p* = 0.002).
- Rotation around the x-axis between method 2 and lower jaw method 3 (*p* = 0.041).
- Rotation around the y-axis between method 2 and lower jaw method 3 (*p* = 0.026), and between upper jaw method 3 and lower jaw method 3 (*p* = 0.041).
- Group 3: A significant difference in translation along the x-axis was found between method 2 and lower jaw method 3 (*p* = 0.013).

These results highlight that in some spatial dimensions, the superimposition methods differ in their accuracy when comparing the upper and lower jaws.

The method error for palatal superimposition in the control group using method 1 was found to be within ±0.3 mm for linear displacements and ±0.8° for rotations based on repeat measurements by independent operators. This confirms the reliability of method 1 for the purposes of this study.

The linear regression analysis, which explored how the variables “age of patients at T0,” “time elapsed between T0 and T1,” and “sex of patients” in the control group affected the agreement between the different superimposition methods, showed a correlation only between the variable “age at T0” and two spatial position variables. The collinearity index (VIF), which measures how strongly the variables are related to each other, had values close to 1 for all measurements. This indicates that there were no collinearity issues between the variables (this applies to all models, as the independent variables were the same in each case). Table 4 displays the linear regression indices (R-squared) and the significance levels for each independent variable.

## 4. Discussion

Clinical orthodontics has always focused on achieving controlled tooth movement in all three planes of space according to the specific treatment plan. Advances in technology now provide orthodontists with increasingly precise tools to evaluate treatment outcomes. One such tool is the superimposition of .stl files, which provides a relatively straightforward method of evaluation.

The purpose of this study was to investigate the differences in superimposition results between two treatment groups. Both G1 and G2 patients underwent non-extraction orthodontic treatment; however, only G2 patients used intermaxillary elastics, which allowed the evaluation of the reliability of the lower arch superimposition during sagittal corrections.

### 4.1. Relevance and Comparison of Superimposition Methods

The results showed that different clinical situations lead to different superimposition results and that different superimposition methods can lead to inconsistent results even for the same clinical condition.

Superimposition of 3D digital models using the palatal surface has been shown to provide accurate and reliable data [19,38,39,40], even over a 5-year period in adolescents [41].

Although the superimposition methods evaluated in this study have inherently different reference areas and serve different purposes, a comparison between them is both valid and essential for several reasons. First, understanding the level of agreement between these methods helps clinicians and researchers make informed decisions about their use in specific contexts. Second, such comparisons allow the reliability of each method to be assessed and its limitations to be identified, ensuring that appropriate methods are used based on desired outcomes. Using quantitative tools such as Bland–Altman plots, this study objectively assesses the discrepancies and acceptable ranges of agreement between the methods, providing practical insights into their clinical and research applications.

### 4.2. Impact of Sagittal Corrections on Superimposition Accuracy

The superimposition methods analyzed in this study revealed notable limitations of best-fit algorithms in patients who underwent sagittal corrections (G2). Specifically, these methods proved less reliable in capturing mandibular positional changes, both in superimpositions of arches locked in occlusion and in individual arch superimpositions, highlighting the reduced accuracy of best-fit algorithms under conditions involving anteroposterior adjustments.

The analysis of patients without planned sagittal corrections (G1) shows that the automatic best-fit superimposition of arches locked in occlusion produces results very comparable to those obtained by palatal superimposition. In particular, the superimposition of arches in occlusion produces more accurate results than the superimposition of individual arches. Specifically, differences were observed in spatial positions along the y-axis (sagittal axis) and z-axis (vertical axis), as well as rotational orientation along the x-axis (roll axis), compared to palatal superimposition. However, rotational orientation along the y-axis (pitch axis) was accurate for both methods 2 and 3, with method 2 (arches in occlusion) demonstrating greater accuracy than method 3 in terms of latero-lateral positioning and rotational alignment.

### 4.3. Clinical Implications of Methodological Differences

The different superimposition methods provide complementary perspectives. Palatal-based superimposition is particularly suitable for detecting global arch stability and growth-related changes, especially in the upper arch.

In contrast, best-fit algorithms are more appropriate for evaluating intra-arch changes, particularly when skeletal growth is minimal or absent, such as in adult patients.

Although Table 2 shows an apparent difference in the measurements obtained using the best-fit superimposition method for the maxilla between G1 and G2, the ANOVA test revealed that this difference was not statistically significant. Conversely, the difference observed between G1 and G2 for the best-fit superimposition of the lower arch was statistically significant. This finding is consistent with the differences in the anteroposterior position of the mandible between the two groups when comparing the single arch palatal and best-fit superimpositions. Similar results were found when comparing the best-fit superimpositions of single arches with those of arches locked in occlusion, further supporting this concept.

From a practical clinical and research perspective, these results suggest that the best-fit superimposition of the maxillary arch closely mirrors the results obtained from superimposition using palatal anatomical references. This is true regardless of whether elastics were used during orthodontic treatment. In other words, automatic best-fit superimposition is a reliable alternative to palatal-based superimposition for upper arch analysis. However, the same cannot be said for the lower arch. Automatic best-fit superimposition of the lower arch is less reliable because it does not take into account changes in the intermaxillary relationship, such as those caused by sagittal corrections or other adjustments to the position of the mandible. This highlights the limitation of using best-fit superimposition for the lower arch, as it may miss important changes in occlusion and jaw alignment. Therefore, more accurate methods—such as palatal reference-based superimposition—should be considered when evaluating mandibular movement, especially in cases where there are changes in intermaxillary relationships. This distinction is critical for both clinical practice and research, as accurate superimposition is essential for evaluating the effectiveness of orthodontic treatment.

### 4.4. Evaluation of Method Reproducibility and Algorithm Limitations

The control group was used to evaluate the reproducibility of the palatal superimposition method (method 1). Ideally, if the dental anatomy remains unchanged between T0 and T1, the automatic best-fit superimposition should produce reliable results [42]. However, measurements in the control group revealed significant differences in anteroposterior positioning when comparing the three superimposition methods. The linear regression analysis showed no correlation between these differences and patient age, sex, or time elapsed between the two intraoral scans. The stability of the palatal reference regions, particularly the medial third of the third palatal rugae and the midpalatal raphe, has been shown to be robust to growth-related changes, thereby mitigating potential confounding from age differences within the sample [41,43]. This suggests that the differences observed in the control group in this study may be due to limitations in the accuracy of the best-fit algorithm used rather than changes in palatal anatomy per se.

The superimposition technique based on palatal ridges has inherent limitations because the palate itself undergoes morphological changes [41,43]. Although this method has been used as a reference for comparison with the other superimposition techniques, it is not the ideal gold standard. A more accurate reference would be superimposition techniques based on stable cranial regions identified by CBCT, which are less susceptible to growth-related changes. The inclusion of CBCT-based superimposition would increase the accuracy and reliability of future studies.

### 4.5. Software Limitations

Our study acknowledges the inherent limitations associated with the use of the Geomagic Control X, 3D Slicer, and Viewbox 4.0 software platforms [44]. Potential sources of error include resolution limitations, alignment discrepancies, and measurement sensitivity in Geomagic Control X; segmentation accuracy and interpolation errors in 3D Slicer; and point marking variability and software-specific limitations in Viewbox 4.0. While efforts were made to standardize workflows and use a single experienced operator to perform all the procedures, the possibility of subtle operator-dependent influences remains. Future studies could address these limitations by incorporating automated and machine learning-based workflows to reduce human variability and by using cross-platform validations to verify results, further increasing the robustness of the results. We minimized these limitations by performing all the landmark placements and analyses by a single experienced operator, standardizing measurement workflows, and using a subset of data to confirm repeatability of the results.

By comparing methods under controlled conditions, this study not only highlights the limitations of best-fit algorithms in certain scenarios, but also underscores the reliability of palatal reference-based superimposition in maintaining stability across a range of clinical applications.

### 4.6. Study Limitations

Possible limitations of the study are as follows:

- The superimposition process on the palatal rugae remains partially operator-dependent, which may introduce variability.
- Age-related anatomical changes, despite being minimized by using stable palatal reference areas, may still influence outcomes, especially in younger patients.
- The absence of CBCT data limits the ability to correlate dental changes with skeletal movements, particularly in cases involving sagittal corrections.
- The inclusion of only patients with Class I and Class II malocclusions. While this decision helped ensure a more homogeneous sample and minimized skeletal variability, it may reduce the generalizability of the results, particularly to Class III cases or in extraction cases. Future research should include a wider range of malocclusion types to better assess the applicability of findings across different clinical conditions.

## 5. Conclusions

This study highlights the importance of selecting superimposition methods based on their distinct applications. For the maxillary arch, palatal-based superimposition is ideal for assessing upper arch stability, growth-related changes, and longitudinal outcomes, due to its reliance on stable anatomical landmarks. In contrast, for the mandibular arch, best-fit algorithms proved more consistent and are more suitable for evaluating intra-arch changes or inter-arch relationships in stable clinical conditions. These findings emphasize that the choice of method should align with the specific clinical or research objectives, providing a practical framework for optimizing orthodontic assessments.

## Figures and Tables

**Table 1:** The Bland–Altman test was utilized to assess the discrepancy between the superimposition methods 2 and 1. The median is the median of the differences between the method of superimposition 2 and 1 (error). The standard deviation of the median difference is represented by σ, and the 95% confidence interval is defined as the minimum and maximum values of the interval. In bold, differences exceeding the threshold values are indicated (σ < 0.25 for x, y, and z; σ< 0.575 for x°, y°, and z°).

Superimposition Method 2 (Best-Fit Archs in Occlusion)								
G1			x	y	z	x°	y°	z°
	median		−0.05	−0.31	−0.24	−0.03	−0.01	0.00
	σ		0.23	**0.68**	**0.42**	**1.43**	0.30	0.54
	95% CI	min	−0.50	**−1.63**	**−1.07**	**−2.84**	−0.60	−1.06
		max	0.40	**1.02**	**0.60**	**2.78**	0.58	1.06
G2			x	y	z	x°	y°	z°
	median		−0.13	−0.46	−0.53	0.22	−0.08	−0.07
	σ		**0.43**	**0.70**	**0.44**	**1.18**	**0.61**	**1.02**
	95% CI	min	**−0.97**	**−1.82**	**−1.40**	**−2.08**	**−1.28**	**−2.07**
		max	**0.70**	**0.91**	**0.34**	**2.52**	**1.13**	**1.93**
G3			x	y	z	x°	y°	z°
	median		−0.02	−0.11	0.01	−0.07	0.05	0.02
	σ		0.09	**0.29**	0.13	0.46	0.20	0.20
	95% CI	min	−0.20	**−0.67**	−0.24	−0.97	−0.33	−0.38
		max	0.17	**0.45**	0.25	0.84	0.43	0.42

**Table 2:** The Bland–Altman test was utilized to assess the discrepancy between the superimposition methods 3 (upper arch) and 1. The median is defined as the median of the differences between the superimposition method 3 (upper arch) and 1 (error). The standard deviation of the median difference is represented by σ, and the 95% confidence interval is expressed as the minimum and maximum values. In bold, the differences exceeding the threshold values indicated (σ < 0.25 for x, y, and z; σ < 0.575 for x°, y°, and z°) are highlighted.

Superimposition Method 3 (Best-Fit Separate Arches: Upper Arch)								
G1			x	y	z	x°	y°	z°
	median		−0.05	−0.05	−0.23	0.29	0.02	0.23
	σ		0.18	**0.81**	**0.43**	**1.49**	0.32	1.35
	95% CI	min	−0.40	**−1.64**	**−1.07**	**−2.63**	−0.61	−2.41
		max	0.30	**1.53**	**0.60**	**3.22**	0.66	2.87
G2			x	y	z	x°	y°	z°
	median		−0.12	0.21	−0.47	0.53	−0.09	−0.21
	σ		**0.39**	**0.74**	**0.52**	**1.24**	**0.58**	**1.33**
	95% CI	min	**−0.89**	**−1.24**	**−1.50**	**−1.90**	**−1.23**	**−2.81**
		max	**0.65**	**1.66**	**0.55**	**2.96**	**1.05**	**2.40**
G3			x	y	z	x°	y°	z°
	median		−0.01	−0.13	−0.01	−0.10	0.01	0.02
	σ		0.10	**0.33**	0.14	0.52	0.20	0.21
	95% CI	min	−0.21	**−0.78**	−0.29	−1.11	−0.39	−0.40
		max	0.19	**0.51**	0.27	0.92	0.41	0.44

**Table 3:** Results derived from the Bland–Altman test, which was utilized for the purpose of comparing the superimposition methods 3 (lower arch) and 1. The median is defined as the median of the differences between the superimposition methods 3 (lower arch) and 1 (error). The standard deviation of the median difference is represented by σ, and the 95% confidence interval is indicated by CI. In bold, the differences exceed the threshold values indicated (σ < 0.25 for x, y, and z; σ < 0.575 for x°, y°, and z°).

Superimposition Method 3 (Best-Fit Separate Arches: Lower Arch)								
G1			x	y	z	x°	y°	z°
	median		−0.08	−0.57	−0.20	−0.69	−0.02	−0.01
	σ		**0.26**	**0.75**	**0.48**	**1.41**	0.32	0.53
	95% CI	min	**−0.60**	**−2.05**	**−1.13**	**−3.45**	−0.64	−1.05
		max	**0.44**	**0.91**	**0.74**	**2.08**	0.61	1.02
G2			x	y	z	x°	y°	z°
	median		−0.25	−1.08	−0.44	0.12	−0.10	−0.50
	σ		**0.77**	**0.82**	**0.52**	**1.91**	**0.76**	**1.51**
	95% CI	min	**−1.77**	**−2.69**	**−1.46**	**−3.62**	**−1.60**	**−3.46**
		max	**1.27**	**0.53**	**0.59**	**3.86**	**1.39**	**2.46**
G3			x	y	z	x°	y°	z°
	median		−0.02	−0.09	0.01	−0.07	0.09	0.00
	σ		0.13	**0.27**	0.13	0.48	0.27	0.23
	95% CI	min	−0.28	**−0.63**	−0.25	−1.01	−0.45	−0.45
		max	0.25	**0.44**	0.28	0.88	0.63	0.46

**Table 4:** Linear regression indices (R-squared) and the significance levels for each independent variable. R Square is a metric of linear regression indices. Sig is the significance coefficient for the relationship specified for each of the independent variables. In bold the statistically significant values (*p* < 0.05).

Control Group (G3)						
	Method 2 (Best Fit. Arch in Occlusion)					
Dependent variables:	x	y	z	x°	y°	z°
R Square	0.4	0.288	0.219	0.297	0.532	0.288
Independent variables	Sig.	Sig.	Sig.	Sig.	Sig.	Sig.
age at T0	**0.008**	0.058	0.572	0.106	**<0.001**	0.178
time between T0 and T1 (days)	0.424	0.233	0.085	0.067	0.65	0.09
Gender = F	0.284	0.896	0.442	0.835	0.696	0.067
	Method 3 sup. (Best Fit. Upper Arch)					
Dependent variables:	x	y	z	x°	y°	z°
R Square	0.293	0.329	0.371	0.379	0.647	0.358
Independent variables	Sig.	Sig.	Sig.	Sig.	Sig.	Sig.
age at T0	**0.027**	**0.033**	0.782	0.064	**<0.001**	0.111
time between T0 and T1 (days)	0.888	0.204	**0.009**	**0.02**	0.077	**0.019**
Gender = F	0.932	0.994	0.237	0.409	0.407	0.282
	Method 3 inf. (Best Fit. Lower Arch)					
Dependent variables:	x	y	z	x°	y°	z°
R Square	0.448	0.238	0.184	0.228	0.276	0.204
Independent variables	Sig.	Sig.	Sig.	Sig.	Sig.	Sig.
age at T0	**0.021**	0.123	0.403	0.083	**0.042**	0.591
time between T0 and T1 (days)	**0.044**	0.285	0.133	0,47	0.941	0.19
Gender = F	0.939	0.711	0.845	0.868	0.808	0.103

## Data Availability

The full anonymized dataset of the study was opened and available from the corresponding author when it is required.
